# Some End Results of Non-Removal of the Appendix in 23 Abscess Cases*Read at the annual meeting of the Medical Association of Georgia, Athens, April 20, 1910.

**Published:** 1910-04

**Authors:** R. M. Harbin

**Affiliations:** Rome, Ga.


### Journal-Record of Medicine

Successor to Atlanta Medical and Surgical Journal, Established 1855.
and Southern Medical Record, Established 1870.

**OWNED BY THE ATLATNA MEDICAL JOURNAL CO.**

**Published Monthly.**

*Official Organ Fulton County Medical Society, State Examining Board, Presbyterian Hospital, Atlanta, Birmingham and Atlantic Railroad Surgeons' Association, Chattahoochee Valley Medical and Surgical Association, Etc.*

EDGAR G. BALLENGER, M. D., Editor.
BERNARD WOLFF, M. D., Supervising Editor.
A. W. STIRLING, M. D., C. M., D. P. H., J. S. HURT, B. Ph., M, D,
GEO. M. NILES, M. D., W. J. LOVE, M. D., (Ala.); Associate Editors.
E. W. ALLEN, Business Manager.

COLLABORATORS

DR. W. F. WESTMORLAND, General Surgery.
F. W. McRAE, M. D., Abdominal Surgery.
H. F. HARRIS, M. D., Pathology and Bacteriology.
E. B. BLOCK, M. D., Diseases of the Nervous System.
MICHEL HOKE, M. D., Orthopedic Surgery.
CYRUS W. STRICKLER, M. D., Legal Medicine and Medical Legislation.
E. C. DAVIS, A. B., M. D., Obstetrics.
E. G. JONES, A. B., M. D., Gynecology.
R. T. DORSEY, Jr., B. S. M. D., Medicine.
L. M. GAINES, A. B., M. D., Internal Medicine.
J. N. LeCONTE, M. D., Diseases of the Stomach and Intestines
L. B. CLARKE, M. D., Pediatrics.
EDGAR PAULIN, M. D., Opsonic Medicine.
R. R. DALY, M. D., Medical Society.
A. W. STIRLING, M. D., Etc., Diseases of the Eye, Ear, Nose and Throat.
BERNARD WOLFF, M. D., Diseases of the Skin.
E. G. BALLENGER, M. D., Diseases of the Genito Urinary Organs.

VOL. LVI. APRIL, 1910 No. 1.

### SOME END RESULTS OF NON-REMOVAL OF THE APPENDIX IN 23 ABSCESS CASES.*

R. M. HARBIN, M. D., ROME, GA.

The question of advisability in prolonging a simple operation for appendical abscess, by removing the appendix and dissecting out the abscess wall, is one that is argued pro and con by competent observers; but it is safe to say that a greater number of surgeons disagree from the radical operation. Prolonged

*Read at the annual meeting of the Medical Association of Georgia, Athens, April 20, 1910.

LIBRARY
SURGEON GENERAL'S OFFICE.
FEB 10 1912
211487

anaesthesia, shock, trauma by manipulations in opening up fresh lymphatic areas, the spreading of infection in an already starved and septic patient, may be mentioned as the chief dangers from the radical operation; while the primary dangers from simple drainage, per se, are nil, with a possibility of recurrent abscess and a longer stay in bed.

Repeated observations on animals have shown a greater susceptibility to the inoculation of bacteria in the peritoneum, under the influence of prolonged anaesthesia, and with other conditions combine to produce shock.

The peritoneum is about equal in area to that of the entire integument of the body, and furnishes an extensive surface for rapid absorption. The peritoneum, however, is capable to taking care of a certain amount of bacteria, and the abundance of exudates proves a decided reaction property to septic processes. If the epithelial covering be impaired or destroyed by mechanical or chemical agents, this reactive power is lost, and septic peritonitis may result. Certain experiments indicate that bacteria can be found in the blood in five minutes after inoculating the peritoneum of rabbits, thus proving the septicemic nature of peritonitis.

While a patient with an appendical abscess has acquired a certain amount of auto-inoculation, local immunity to the harbored infection, at the same time his powers of resistance are lessened because of a starved and septic condition, and is less capable of resisting any fresh bacterial invasions. The rapidity of escape, as well as the virulence of the bacteria determines whether the case will be one of abscess or one of a perforative and spreading type of peritonitis.

It is easily understood why the exudate remains of a previous attack, in many instances hinders the development of a diffuse peritonitis, and would tend to the formation of an abscess. In this series of consecutive cases, seven had previous attacks, while sixteen were primary. The dangers from incision and drainage are slight, because few fresh areas for absorption are opened up, and instead of the marked reactionary symptoms following the radical operation, we should have a lessened pulse rate and lower temperature by relieving the tension of the parts affected.

It is perhaps more satisfactory to feel, when the patient leaves the operating table, that he will be rid of any recurrent troubles, but such a feeling may be bought by many hazards. Of course, the appendix should be removed when it can be done without undue manipulation or dissection. I have had a series of abscess cases in which the appendix was easily removed, but are not included in this report. When the appendix can be removed a burden of sepsis will be removed, especially in those cases in which the abscess is immature.

We are confronted with a choice between the dangers of sepsis from a retained appendix on the one hand, and the risk of spreading peritonitis by its removal, on the other hand; and we must bear in mind that post-operative peritonitis is more dangerous than the spontaneous variety of the disease. Of course individual conditions should determine the proper course to pursue, and are not always clear. Where drainage has been established, the danger from a sloughing appendix protected by adhesions, is one of sepsis and not peritonitis, and depends on the virulence of the infective bacteria. If the type of infection be one of unusual virulence, an extensive dissection probably cannot be done in an aseptic manner, and will only aggravate the existing condition, while in other cases satisfactory results may be obtained by a gauze pack drain, leaving the process to the active reparative power of the peritoneum. I have frequently seen an almost normal temperature and pulse throughout from this procedure. The few records of findings at autopsies of such cases show almost complete disappearance of the vestiges of the previous suppurative processes. The case is recalled of a perforated appendix imbedded on the right side of caput coli, and appearing at the bottom of the incision, was enucleated. Thirty minutes after the operation there occurred a severe rigor, followed by death in twenty-four hours. Life may have been prolonged if only packing had been done. All of these were late cases and no active search was made for the appendix.

This series of cases includes only those in which pus accumulated in the region of the appendix, protected by an abscess wall. The technique of operations consisted of short incisions, few manipulations, loose gauze drainage, supplemented by postural methods, such as that of Fowler, combined with the dorsal, left lateral and ventral positions, and proctoclysis of salt water. There were six, or 26 per cent. cases of the parietal form of abscess. In other than parietal forms of abscess, the incisions were made below and to the right of the abscess, so as to invade the mass from behind, in order to make a portion of the iliac fossa a part of the sinus wall.

Prolonged drainage seemed to prevent recurrence, and with a short and low incision, does not increase the tendency to hernia. Various surgeons estimate the recurrences from one to fifty per cent, and these operations lead me to suspect that such a variety of results is to be accounted for by a difference of technique.

There were three, or 13 per cent., that gave recurrent troubles, but none were of serious import. In a child, the sinus discharged intermittently for eight months, and no recurrence has appeared in three years, and in another, a man thirty-five years old, the discharge continued six months, without incapacitating him for work, and has remained well two years. In another, the retro-peritoneal abscess appeared in the lumbar region, and was drained there, but was reserved for another operation, when he disappeared from observation. There were no cases of fecal fistulae. From a series of 96 cases of non-removal of the appendix by Clogg (in Lancet Mch. 6, 1909) the percentage of recurrent troubles was estimated at 43—divided as follows: sinus 18 per cent., recurrent appendical 11 per cent., suppuration, 8 per cent., suphraical abscess, 3 per cent., protal sepsis, 1 per cent.

Sixteen were operated in the remote rural districts under unfavorable conditions, among the laboring class, who would likely give troublesome sequelae. One case had hernia after twelve months, and only one before that period. The average age was 16.6 years, youngest 4 and oldest 58 years. There were sixteen males and seven females. The longest time of attack before operation was nineteen, the shortest three and average eight days. Peculiar locations of abscesses were noted in two cases, one appearing in the right lumbar region, and another in the middle line and adherent to the bladder. In two cases the abscess burrowed through the layers of the abdominal wall. The longest stay in bed was 30 and the shortest 20, and average 23.8 days. This series of cases covers a period of ten years in which sufficient time has elapsed to determine final results. All are now living and strong except one woman who died from pulmonary tuberculosis one year after operation. There was one death or 4.3 per cent. from unabated sepsis after 28 days.

*Conclusions.*

1. The choice lies between the danger of sepsis from a retained appendix, with a possibility of recurrence and longer stay in bed, on the one, from simple drainage: and that of spreading peritonitis on the other hand from the radical operation with its immediate dangers.

2. The usually starved and septic condition of the patient with an appendical abscess does not warrant a radical operation.

3. The remarkable reparative powers of the peritoneum argue for the simple operation.

4. The suggestive value of this limited number of cases warrants the belief that recurrences after the simple operation are rare and still more rarely serious.

5. There was a mortality of 4.39.

